# Potential for local adaptation in response to an anthropogenic agent of selection: effects of road deicing salts on amphibian embryonic survival and development

**DOI:** 10.1111/eva.12016

**Published:** 2012-10-01

**Authors:** Gareth R Hopkins, Susannah S French, Edmund D Brodie

**Affiliations:** Department of Biology and the Ecology Center, Utah State University LoganUT, USA

**Keywords:** amphibian, egg, local adaptation, magnesium chloride, natural selection, road deicing salt, *Taricha granulosa*, variation

## Abstract

The application of millions of tons of road deicing salts every winter in North America presents significant survival challenges to amphibians inhabiting roadside habitats. While much is known of the effects of NaCl on anuran tadpoles, less is known of effects on amphibian eggs, or any caudate life stage. In addition, little is known of the effects of MgCl_2_, which is now the 2nd most commonly used road deicer. Most studies have considered amphibians to be helpless victims of deicing salts, and ignore the possibility of the evolution of local adaptation to this stressor. We attempt to address these knowledge gaps and explore this evolutionary potential by examining the effects of NaCl and MgCl_2_ on the survival and development of eggs from different female rough-skinned newts (*Taricha granulosa*) from the same population. We demonstrate that both salts, at environmentally relevant concentrations, severely affect the embryonic survival and development of this amphibian, but that the effects of the salt are dependent on the identity of the mother. This female × treatment interaction results in substantial variation in tolerance to road deicing salts among newt families, providing the raw material necessary for natural selection and the evolution of local adaptation in this amphibian.

## Introduction

Roads are a dominant feature of the North American landscape, with approximately the same coverage area as streams (∼1% of the land area of the contiguous United States; Forman 2000; Riitters and Wickham 2003). It has been estimated that 20% of the total land area of the contiguous United States is located within 127 m of a road (with only 3% located over 5 km away) (Riitters and Wickham 2003), and this proximity has been shown to have direct and indirect ecological consequences (Forman 2000; Trombulak and Frissell 2000). These include impacts on species richness [e.g., with amphibians (Houlahan and Findlay 2003; Collins and Russell 2009)], mortality from construction and vehicle collision, the spread of exotic species, and the alteration of the physical and chemical environment (reviewed by Trombulak and Frissell 2000).

One of the major ways in which roads alter the chemical environment of ecosystems is through the runoff of contaminants, including heavy metals, organic pollutants, and road deicing salts. An estimated 14 million tons of deicing salts are applied every winter in North America (Environment Canada 2001). This application has arguably led to the dramatic salinization of fresh water in areas of the continent over the last three decades (Kaushal et al. 2005). Chronic exposure to chloride concentrations greater than 240 mg/L has been deemed harmful (median lethal concentration) to approximately 10% of aquatic life (Environment Canada 2001; Kaushal et al. 2005), and concentrations in large excess of that limit, up to 4000 mg/L Cl^−^, have been found in roadside ponds and wetlands today (Environment Canada 2001). Chloride concentrations in roadside ponds have been found to greatly exceed those in ponds located away from roads (Turtle 2000; Karraker et al. 2008; Collins and Russell 2009; Brady 2012). Traditionally, NaCl has been the primary component of road deicers. More recently, however, other deicers, such as MgCl_2_, have been used alongside or in place of NaCl, due to their increased performance at low temperatures and decreased corrosive properties (Forman et al. 2003; Harless et al. 2011). According to a recent survey of 22 states and three Canadian provinces by the National Transportation Research Board (2007), liquid MgCl_2_ is currently the 2nd most widely used chemical deicer in North America, after NaCl. Although NaCl may be the most abundantly applied salt, a study examining the leaching of road deicing salts in soils in New York State found that Mg^2+^ from MgCl_2_ was the most abundant, reactive salt cation in roadside soils (Cunningham et al. 2008). In addition, MgCl_2_ is now being used exclusively by some agencies, in place of NaCl (e.g., Oregon Department of Transportation 2012; Kendal Weeks, Oregon Department of Transportation Road Maintenance, personal communication). Despite this prevalence of use, very few studies have examined the potential biological effects of this deicing salt on the inhabitants of freshwater ecosystems.

Amphibians, with their permeable skin and eggs, are an osmotically challenged group of animals (Shoemaker and Nagy 1977). Adult tree frogs, for instance, have been recorded to lose, through evaporative passive water flux, up to 250 mL body water/kg/day, far exceeding the less than 3 mL/kg/day loss of most reptiles (Shoemaker and Nagy 1977). At the same time, most freshwater amphibians have evolved to pump ions including Na^+^ and Cl^−^ into their bodies because they live in hypo-osmotic environments (Duellman and Trueb 1994). These adaptations for acquiring and retaining ions, not for removing or restricting them, combined with the constant challenge of retaining water in the body cavity, mean that amphibians are particularly maladapted to hyperosmotic environments, such as salt water. The survival and demography of all life-history stages of frogs and toads, specifically, have repeatedly been shown to be negatively affected by NaCl (Padhye and Ghate 1992; Viertel 1999; Dougherty and Smith 2006; Karraker et al. 2008; Collins and Russell 2009; Karraker and Ruthig 2009; Langhans et al. 2009; Petranka and Doyle 2010; Harless et al. 2011; Alexander et al. 2012), and two recent studies suggest that MgCl_2_ may actually be more toxic than NaCl to tadpoles (Dougherty and Smith 2006; Harless et al. 2011). Most studies on the effects of salt on amphibians have focused on the adult or larval stage, ignoring the eggs, despite some evidence suggesting that eggs may in fact be the most susceptible life-history stage to salt (Beebee 1985; Padhye and Ghate 1992; Karraker and Ruthig 2009). In addition, nearly all studies have been conducted on anuran amphibians, despite the published assertion (Neill 1958) and field and experimental evidence (Karraker et al. 2008; Collins and Russell 2009; Karraker and Ruthig 2009; Chambers 2011) that salamanders and newts (i.e., caudates) may actually be more sensitive to salt than frogs and toads. We address these gaps in our knowledge, in this paper, by examining the effects of both NaCl and MgCl_2_ road deicing salts on the survival and development of eggs from the rough-skinned newt (*Taricha granulosa* Skilton), a common salamandrid amphibian inhabiting the west coast of North America, an area where both NaCl and MgCl_2_ are currently used (Warrington 1998; California Department of Transportation 1999; Oregon Department of Transportation 2012; Washington State Department of Transportation 2012).

In response to environmental stressors such as road deicing salt, amphibians have traditionally been viewed as helpless players, and thus, the potential for local adaptation and the evolution of tolerance to the stressor has largely been ignored (Brady 2012). However, we are now aware that evolution can act on ecological time scales and in response to anthropogenic stressors (Carroll et al. 2007) and that local adaptation via natural selection can mediate responses to contemporary environmental change (Kawecki and Ebert 2004). Indeed, the moor frog, *Rana arvalis*, has been shown to locally adapt to acidification of its habitat (Andrén et al. 1989; Merilä et al. 2004), and recently, Brady (2012) showed that populations of the salamander *Ambystoma maculatum* appear to be locally adapted to living adjacent to roads. This local adaptation was attributed to a possible evolved tolerance of road deicing salts. The underlying basis for local adaptation is natural selection (Kawecki and Ebert 2004), and so it is first important to demonstrate that the raw material for selection, namely variation in adaptive traits, is present in a population if one is to understand the potential for local adaptation to occur in response to a stressor. Understanding this potential, and the mechanisms behind such evolution of salt tolerance, is critically important for conservation efforts (Carroll et al. 2007), especially in this era of unprecedented declines in amphibian populations worldwide (Stuart et al. 2004; Mendelson et al. 2006).

The purpose of our study was twofold: (i) to determine the effects of two commonly used road deicing salts, NaCl and MgCl_2_, on the embryonic survival and development of *Taricha granulosa* and (ii) to investigate the possibility for the evolution of local adaptation in this species, by determining whether significant variation in salt tolerance exists among families of newts from a single population.

## Materials and methods

### Experimental animals

Sixteen gravid adult female rough-skinned newts (*Taricha granulosa*) were collected by hand and dip-net from the Soap Creek ponds in Benton County, Oregon (44°40′14.10″N, 123°16′37.47″W), April 4–5, 2011, for use in this study. These ponds are a series of eight rectangular (22.86 × 91.44 m, 3–4 m deep) man-made ponds, in two rows of four, each pond separated from the next by only a 2-m grassy berm (Gall et al. 2011a). This collection of closely spaced ponds are surrounded by oak woodland and feral pasture and together represent an environmentally homogenous habitat, home to a single population of newts (Gall et al. 2011a). *Taricha granulosa* have empirically been shown to be highly philopatric to these ponds (Landreth and Ferguson 1967), and there is no appreciable genetic structuring of newt populations in this geographic area (within at least 20 km of the ponds) (Jones et al. 2001). The Soap Creek ponds are located 313 m away from the nearest paved road (a small, two lane, county road) and are separated from the road by several areas of low-elevation land, which would prevent runoff from the road from reaching the ponds. Deicing salts are not applied to this road, or any of the county roads in this area (Kendal Weeks, Oregon Department of Transportation Road Maintenance, personal communication; Kent Mahler, Benton County Road Maintenance, personal communication). Salt (MgCl_2_) is also not applied to the stretch of highway closest to the ponds (Kendal Weeks, Oregon Department of Transportation Road Maintenance, personal communication), which is located over 4 km away, at a similar elevation, and the intervening area is covered with a series of hills not conducive to a flow of runoff from the highway reaching the ponds.

All animals were brought back to Utah State University where they were housed individually in 37.85-L glass aquaria with approximately 14 L of filtered, chilled tap water. Newts were housed in an environmental control chamber at 7°C on a 12-h:12-h light/dark cycle and fed blackworms (*Lumbriculus variegatus*) *ad libitum* prior to commencement of experiments. Methods adapted from Hopkins et al. (2012) throughout.

Females were injected with 10 μL luteinizing-hormone-releasing hormone ([des-Gly10, D-His(Bzl)6]-LHRH ethylamide; Sigma #L2761) to induce egg deposition and provided a small piece of polyester fiber and branch as oviposition sites. All females began depositing eggs during April 10–15 and were finished depositing by April 22–May 9. All eggs were collected and separated from the oviposition site within 12 h (at which point timing of the lengths of the embryonic period began) and stored in plastic containers with chilled tap water before being assigned treatments. After all eggs were deposited, the mass and snout-vent length (SVL) of all females were recorded. Egg diameter was measured for 10 eggs per female (eggs not used in the experiment) using an ocular micrometer with an Olympus stereomicroscope (Olympus America, Center Valley, PA, USA).

### Solution preparation

Newt eggs were reared in seven different solutions: six treatments, and one control. Control solution was composed of 20% Holtfreter's solution, a solution recommended for the successful development of caudate embryos (Armstrong et al. 1989), which corresponds to a salinity of 0.7 g/L Cl^−^. Treatment solutions were low (1.0 g/L Cl^−^), medium (1.5 g/L Cl^−^), and high (2.0 g/L Cl^−^) concentrations of NaCl and MgCl_2_, made by mixing pure biological crystalline laboratory-grade salts (Thermo Fisher Scientific (Fair Lawn, NJ, USA) – NaCl and Acros Organics (Fair Lawn, NJ, USA) – MgCl_2_) with distilled H_2_O. These salt concentrations are well within realistic field limits (of up to 4.0 g/L Cl^−^) that have been reported in roadside ponds in North America (Environment Canada 2001), and the Cl^−^ concentrations (up to 2.05 g/L) reported for runoff from salted roads into aquatic habitats in parts of *Taricha granulosa*'s range (Hoffman et al. 1981). Small amounts of buffer were added to solutions if necessary to ensure the pH of all solutions was approximately neutral (7.0–7.5). All solutions were stored in sealed glass jars at 7°C in the same environmental chamber as the newts until the commencement of experiments.

### Experimental procedure

Within 12 h of oviposition, each female's eggs were randomly assigned in groups of three to a lidded round plastic petri dish (hereafter ‘cup’) (3.5 cm diameter, 1 cm deep), glued to a plastic cafeteria tray (40 × 31 cm) for stability, which was itself assigned to a random location within a 2.6 × 3.1 × 2.3 m environmental control chamber at 7°C with a 12-h light/12-h dark cycle. Each cup was randomly assigned either the control solution or one of the six experimental treatments. Four milliliters of this solution was pipetted into each cup, and three of the assigned female newt's eggs were carefully placed in the corresponding cup. Eggs from different females were never mixed in the same cup. A line was drawn on the outside of each cup at this point to indicate the water level and cups were checked on a daily basis throughout the experiment for dehydration. If needed, a small amount of distilled H_2_O was added to cups to bring the water level up to this line again; adding distilled water only ensured that the salinity concentration of each cup remained constant throughout the experiment, as water might have evaporated, but the salt in the solution would have remained. Each cup was labeled with a unique number as well as the assigned female and treatment and date of oviposition. All eggs were deposited during April 15–May 9, 2011. Female newts deposited between 117 and 594 eggs each (mean ± SE = 300.13 ± 36.02), corresponding to a total of 2577 eggs placed in control (859 cups), 363 eggs in low NaCl (121 cups), 369 eggs in low MgCl_2_ (123 cups), 366 eggs in medium NaCl (122 cups), 369 eggs in medium MgCl_2_ (123 cups), 345 eggs in high NaCl (115 cups), and 354 eggs in high MgCl_2_ (118 cups). Assignment of eggs was weighed more heavily to control, as more animals were needed for a separate experiment on larval biology (not discussed here).

Cups were checked on a daily basis for dead eggs, which were noted and removed, keeping track of what day the egg died, who its mother was, and what treatment and cup number it was raised in. Cups were also monitored on a daily basis for egg hatching, and when hatchlings were free-swimming, the date of hatching was recorded, and the hatchling was removed from its cup with a pipette. The total length of each hatchling newt was then immediately measured to the nearest millimeter using an ocular micrometer attached to an Olympus stereomicroscope, and its developmental stage (Harrison 1969) was recorded. Although SVL is typically measured in wild caudate populations because of the overriding issue of tail autotomy, for newly hatched newt larvae in the laboratory, not facing this issue, measuring the total length of hatchlings is considered a more appropriate estimate of body size.

### Statistical analysis

To calculate the time eggs were alive, we subtracted the date eggs were deposited from the date eggs died (for eggs that died) or the date eggs hatched (for eggs that survived). For the survival data, the total number of eggs that died in each cup compared to the total number of eggs per cup was tabulated and used as the number of ‘events’ per ‘trial’ in a logit-linked generalized linear model.

All analyses were conducted on egg-level data, with the random structuring effect of cup included in the model. For the normally distributed variables of time eggs were alive (days), hatching timing (days), developmental stage at hatching, and size at hatching (mm), we used a generalized linear mixed model with two-way anovas and Type III sums of squares in PROC GLIMMIX in SAS®, with the fixed-effect factors of salt treatment, individual female, and their interaction. These factors were also nested within cups as a random-effect factor to account for this experimental structuring of the egg-level data. While individual female is often thought of as a random factor in analyses, we chose to treat it as a fixed-effect factor, as we were specifically interested in how the mean responses of these specific, representative females differed from each other in regard to salt tolerance. Analyzing this sort of dependency of individual female identity on the effect of salt treatment on eggs involves the creation of an interaction term that is only possible when both salt treatment and female are treated as fixed, not random effects (Bennington and Thayne 1994). We analyzed the egg survival data using logistic regression with PROC LOGISTIC in SAS®, and Type III tests of fixed effects. We applied the Williams method (Williams 1982) to address overdispersion caused by eggs nested within cups, and a penalized maximum-likelihood estimation ‘Firth’ correction (Firth 1993; Allison 2008) to deal with issues of quasi-separation of data caused by some cups having 100% survival or mortality. If there was an overall significant effect of treatment, we conducted Tukey-adjusted multiple comparison tests between all treatment levels to determine the effect of the different salt concentrations on all variables.

If individual female was found to have a significant effect on any of the response variables, we reanalyzed our normally distributed data to see whether any visible morphological characteristics of the females could explain the interfamily variation we observed. Female mass, SVL, mean egg diameter, and the total number of eggs laid were incorporated individually into a mixed ancova model with the morphological trait in question treated as a continuous fixed-effect factor and treatment as a categorical fixed-effect factor. In this model only, female as a fixed-effect factor is replaced by morphological trait as a continuous fixed-effect factor, and female is treated as a random factor, because female identity and morphological trait of that female are completely confounded with each other when both are treated as fixed in the same model. Of specific interest in each of these trait models is the treatment by trait interaction, the significance of which could indicate that a given maternal characteristic is implicit in the significance of the female by treatment interaction in our original models.

All statistical analyses were conducted using SAS® software version 9.3 (SAS Institute Inc., Cary, NC, USA), with significance set at α = 0.05.

## Results

**Figure 1:**
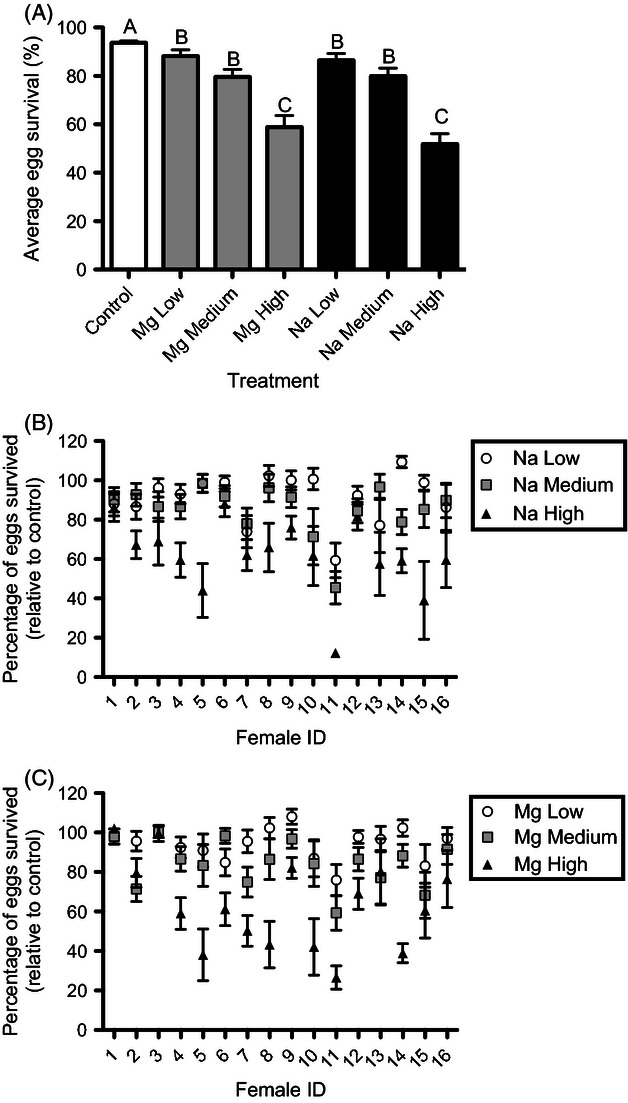
The effect of salinity concentration (A) and maternal identity (B, C) on egg survival raised under increasing concentrations of NaCl (A, B) and MgCl_2_ (A, C). (A) There is a significant effect of salt treatment on mean (±SE) egg survival in each treatment (Table 1 for detailed statistics). Different letters indicate significant differences between treatments (Tukey-adjusted multiple comparisons). (B) Mean (±SE) percentage of eggs survived in three increasing concentrations of NaCl relative to the survival of those eggs raised under control conditions for 16 different female newts. There is significant variation in the response of eggs from different females to the treatments (Table 1 for detailed statistics). (C) Same results for three increasing concentrations of MgCl_2_.

There was a significant overall effect of salt treatment, female identity, and the interaction between treatment and female for all response variables measured (Table 1). There was significant underlying variation among females in all variables at the control level (Figure S1), and so, for illustrative purposes, the effects of salt treatments on individual females are displayed relative to effects seen at the control level (Figs 1B, C and S2).

**Table 1:** The overall effect of salinity treatment, individual female, and the interaction between treatment and female on egg survival (logistic regression), time eggs alive (days), time to hatching (days), developmental stage at hatching, and size (length – mm) at hatching (two-way anovas)

	Egg survival		Time egg alive		Time to hatching		Stage at hatching		Size at hatching	
					
	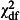	*P*	*F*_df_	*P*	*F*_df_	*P*	*F*_df_	*P*	*F*_df_	*P*
Treatment	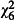 = 198.03	<0.0001	*F*_6,1469_ = 61.11	<0.0001	*F*_6,1367_ = 64.55	<0.0001	*F*_6,1366_ = 346.69	<0.0001	*F*_6,1367_ = 455.15	<0.0001
Female	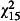 = 139.82	<0.0001	*F*_15,1469_ = 5.36	<0.0001	*F*_15,1367_ = 3.79	<0.0001	*F*_15,1366_ = 6.15	<0.0001	*F*_15,1367_ = 9.30	<0.0001
Female× treatment	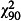 = 115.52	0.0363	*F*_90,1469_ = 2.48	<0.0001	*F*_89,1367_ = 1.95	<0.0001	*F*_89,1366_ = 2.65	<0.0001	*F*_89,1367_ = 2.18	<0.0001

### Treatment effects

**Figure 2:**
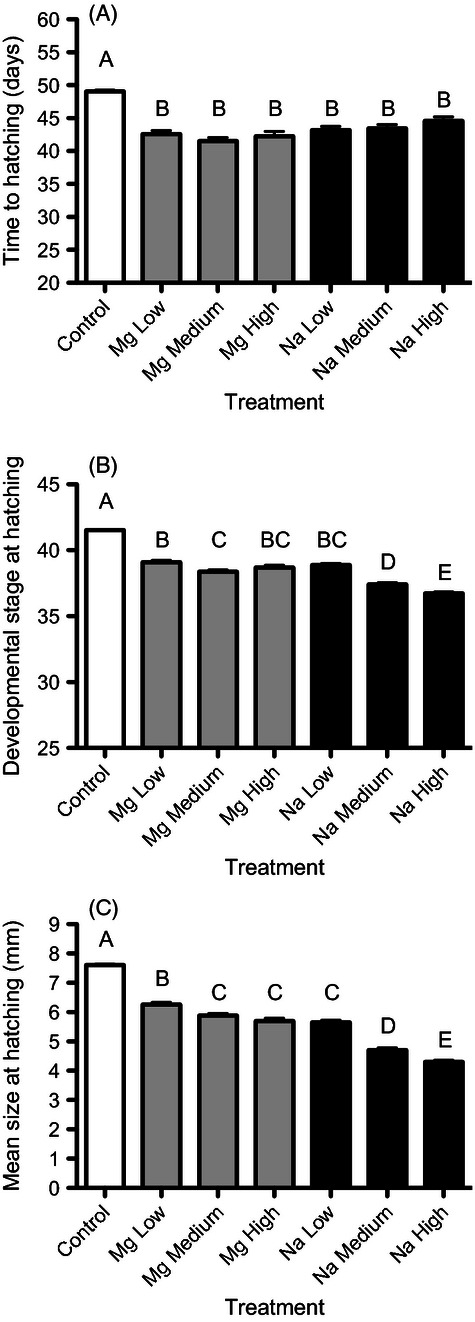
The significant effect of salt (NaCl and MgCl_2_) concentration on mean (±SE) (A) Time to hatching (days). (B) Developmental stage at hatching (Harrison 1969) and (C) Mean size (total length – mm) at hatching. Different letters indicate significant differences between treatments (Tukey-adjusted multiple comparisons).

Increasing concentrations of either NaCl or MgCl_2_ in general caused more eggs to die (Fig. 1A) and sooner (Figure S3A). Those eggs that survived the treatment generally hatched out sooner in salt treatments (Fig. 2A), were less developed (Fig. 2B) and smaller (Fig. 2C). For all variables, control eggs fared significantly better than eggs in any of the salt treatments (Figs 1A and 2). Egg survival responded to increasing salt concentration in a dose-dependent fashion, and there were no significant differences between the effects of NaCl and MgCl_2_ (Fig. 1A). Eggs survived longer in NaCl versus MgCl_2_ at high concentrations, but the effects at low concentrations were similar between the two salts (Figure S3A). All salt treatments caused premature hatching relative to eggs raised in control, but there did not appear to be a specific dose-dependent relationship among salt treatments, and MgCl_2_ and NaCl caused statistically similar effects. High and medium NaCl treatments caused larvae to hatch slightly smaller and less developed than those raised in MgCl_2_, but the effects of the two salts at low concentrations were very similar (Fig. 2B, C).

### Female effects

There was significant, underlying variation in egg survival, time eggs were alive, time to hatching, and developmental stage and size at hatching among eggs from different females (‘families’) raised in control solution (Figure S1), and this variation persisted under salt treatments (Figure S2). The variation among families interacted with treatment such that the magnitude of the effect of salt treatment on eggs depended on which family the animal belonged to (significant female × treatment interaction term, Table 1). The variation among families is demonstrated in Table S1 by showing the minimum, maximum, and mean values for families at different treatments, with an increasing trend in interfamily variation with salt concentration.

None of the interactions between morphological female trait and salt treatment were statistically significant at *α* = 0.05 (with the exception of time to hatching as a function of egg diameter), indicating that the morphological measured traits of the females probably played a relatively small part in accounting for the significant interfamily variation and female × treatment interaction we observed in our original models.

## Discussion

Road deicing salts, at environmentally relevant concentrations, caused significant mortality of newt eggs in a dose-dependent fashion. Those salt-exposed eggs that survived hatched out sooner and were less well developed and smaller compared to control animals. There was little difference in the overall effects of NaCl and MgCl_2_, indicating that MgCl_2_, an emerging stressor, is at least as toxic to *Taricha granulosa* eggs as the more traditionally used NaCl. There was significant variation among newt families within a single population in the effects of salt on all variables examined, with some families exhibiting extreme tolerance to salt (e.g., 84–100% survival in high salt concentrations) and others severe susceptibility (e.g., 0% survival in high salt concentrations). It appears that the raw material for natural selection to act upon is present for the evolution of local adaptation to road deicing salts in *T. granulosa*.

All life stages of amphibians have been shown to be severely affected by road deicing salts, with increased concentrations of NaCl causing egg, larval and adult mortality and impaired growth and development (Viertel 1999; Turtle 2000; Dougherty and Smith 2006; Karraker 2007; Collins and Russell 2009; Karraker and Ruthig 2009; Langhans et al. 2009; Petranka and Doyle 2010; Duff et al. 2011; Harless et al. 2011; Alexander et al. 2012), similar to results found for *T. granulosa* eggs in this study. The effect of MgCl_2_ on amphibians is less well understood, despite its now prevalent use (National Transportation Research Board 2007; Cunningham et al. 2008). We found that MgCl_2_ was, in general, at least as toxic as NaCl. Only two recent studies (Dougherty and Smith 2006; Harless et al. 2011) have examined MgCl_2_ toxicity on any life stage of amphibian, and their results also indicate that MgCl_2_ may be at least, or even more, toxic to some frog tadpoles than NaCl.

Increased concentrations of both road deicing salts caused increased *T. granulosa* egg mortality, as has been found for NaCl in many other amphibian species (Beebee 1985; Padhye and Ghate 1992; Viertel 1999; Turtle 2000; Karraker et al. 2008; Karraker and Ruthig 2009; Petranka and Doyle 2010; Alexander et al. 2012). However, even those eggs that survived to hatching did not escape the negative effects of increased salt concentrations. Salt treatments caused *T. granulosa* embryos to hatch earlier, at a less-developed stage and a smaller size, similar to anuran embryos exposed to NaCl (Ruibal 1959; Padhye and Ghate 1992). In some cases, we observed hatchlings that had not fully developed morphologies, presenting serious survival liabilities. However, larvae hatching with all essential structures fully developed, but earlier, or at a smaller size, may still have significant fitness consequences in relation to survival, the onset of feeding competence, and competitive and predatory interactions (Warkentin 1995, 1999; Boone et al. 2002). For example, smaller, less-developed *T. granulosa* larvae are more likely to be injured and die as a result of predatory attacks by dragonfly nymphs (Gall et al. 2011b).

To our knowledge, effects of road deicing salts on caudate embryonic survivorship have only been examined in one other species, the spotted salamander, *Ambystoma maculatum* (Turtle 2000; Karraker et al. 2008; Karraker and Ruthig 2009; Karraker and Gibbs 2011; Brady 2012). Similar to our results with *T. granulosa*, *A. maculatum* embryonic survival significantly declines with increasing road salt (NaCl) concentration (Karraker et al. 2008; Karraker and Ruthig 2009), and NaCl concentrations approximately analogous to our ‘low’ salt concentrations (∼1.0 g/L Cl^−^) have been found to permanently disrupt the osmoregulatory ability of *A. maculatum* eggs (Karraker and Gibbs 2011). Results with *A. maculatum* suggest that this species may be more susceptible to salt than anuran amphibians (Karraker et al. 2008; Collins and Russell 2009; Karraker and Ruthig 2009), and our results support the assertion that caudates in general may be more sensitive to salt than anurans (Neill 1958). We found severe, negative effects of salt on *T. granulosa* embryos at concentrations of 2.0 g/L Cl^−^, whereas studies on anuran eggs have not found negative effects until concentrations of over 4.0 g/L Cl^−^ are reached (e.g., Petranka and Doyle 2010; Alexander et al. 2012). Studies on *A. maculatum* by Turtle (2000) and Brady (2012) did not directly test the effects of road deicing salts, but rather compared survival in roadside versus woodland ponds, and correlated this with field measurements of increased salinity in roadside ponds. While both authors found that embryonic survival decreased significantly in roadside compared to woodland ponds, Brady (2012) also found that *A. maculatum* eggs naturally occurring in roadside ponds survived better in this environment than eggs transplanted there from woodland ponds, indicating that local adaptation to this environment may have occurred. Linking this adaptation to tolerance of road deicing salt, specifically, is, however, only correlative.

The driving force behind local adaptation is natural selection (Kawecki and Ebert 2004). In order for the selection regimes necessary for the evolution of local adaptation to operate, rare alleles must exist that improve fitness in a habitat in which most individuals perform poorly (Kawecki and Ebert 2004). Our results show that environmentally relevant concentrations of salt are able to kill up to 100% of the offspring of many female newts, while the offspring from other females experience 100% survival in this same salt concentration. For alleles promoting tolerance to exist, such variation must be under selective pressure (Kawecki and Ebert 2004). We have previously established that newt early life-history traits have the potential to be under selective pressure, in possessing the underlying interfamily variation necessary for natural selection (Hopkins et al. 2012), and we confirm and elaborate on this finding here. In the present study, we show that road deicing salt is able to act as a force of selection, by demonstrating significant interfamily variation in survival and embryonic development in response to increased concentrations of salt. We have further determined that a significant interacting effect between female genotype and saline environment exists for newt fitness, which is another prerequisite for local adaptation (Kawecki and Ebert 2004).

To what extent the variation we observed is entirely genetic, and thus able to lead to evolutionary change, remains to be determined, as we were unable to establish paternity in this study. We also cannot absolutely discount unmeasured nongenetic maternal effects such as differences in female lipid, hormone content, or yolk quality (e.g., Crump and Kaplan 1979) from contributing to the observed variation. We did try to account for some maternal effects via estimating the effects of maternal body size and weight, clutch size, and egg diameter on the observed variation. We found, similar to Brady (2012), that the measured maternal influences did not greatly influence embryonic survival or development.

Despite not knowing the extent to which the observed variation is genetic, ours is, to our knowledge, the first study to examine interfamily variation in an amphibian's tolerance to road deicing salt, which in itself is an important prerequisite for natural selection leading to the evolution of local adaptation. Fully determining the genetic nature of this variation and comparing the fitness of newt populations inhabiting roadside and non-roadside ponds (Brady 2012) are obvious next steps in determining whether local adaptation has indeed occurred in this amphibian species. Fully understanding and exploring this evolutionary potential is critical for conservation efforts (Carroll et al. 2007) in this time of unprecedented declines in amphibian populations worldwide (Stuart et al. 2004; Mendelson et al. 2006), and the increasing salinization of freshwater resources (Thunqvist 2004; Kaushal et al. 2005) due to an ever-expanding network of roads across the landscape (Forman 2000; Forman et al. 2003; Riitters and Wickham 2003).

## Data archiving statement

Data for this study are available at Dryad: doi: 10.5061/dryad.qn24m

## Supporting Information

**Figure S1.** The interfamily variation in (A) percentage of eggs survived, (B) time eggs survived (days), (C) time to hatching (days), (D) developmental stage at hatching, and (E) size (length) at hatching (mm) for the offspring of 16 different female newts raised in Control solution. (C-E) Reprinted from Hopkins et al. (2012).

**Figure S2.** Significant variation in the response of eggs from different females to NaCl (A, C, E) and MgCl_2_ (B, D, F) salt concentrations for (A, B) Time to hatching (days before eggs hatching in Control), (C, D) Developmental stage at hatching (number of developmental stages below larvae hatching in Control), and (E, F) Size (total length in mm) at hatching (mm below the size of larvae hatching in control).

**Figure S3.** The effect of salinity concentration (A) and maternal identity (B, C) on time eggs survived (days) raised under increasing concentrations of NaCl (A, B) and MgCl_2_ (A, C).

**Table S1.** Variation among families in (a) egg survival, (b) time to hatching (c) stage at hatching and (d) size (length) at hatching at different treatments, reporting the mean, minimum and maximum values (mean ± SE) for families for each treatment.
