# Supplementary figures and images for: Potential for local adaptation in response to an anthropogenic agent of selection: effects of road deicing salts on amphibian embryonic survival and development
Source: Evol Appl. 2012 Oct 1;6(2):384–92. doi: 10.1111/eva.12016 (PMC3586626; doi:10.1111/eva.12016)

**Figure 1**  
**Supplemental Information**

**A.**

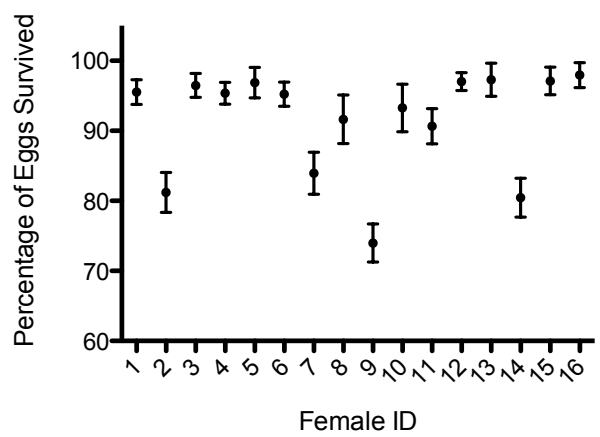

**B.**

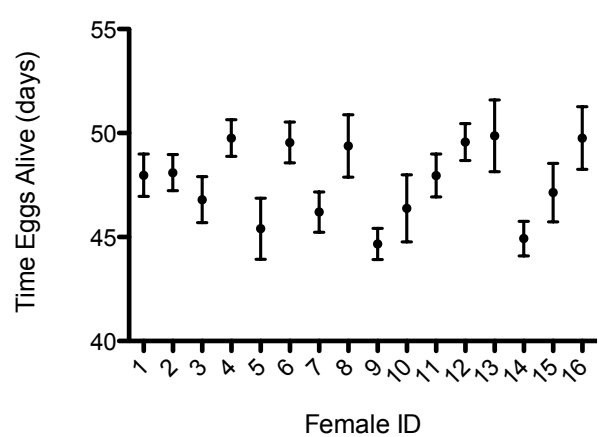

**C.**

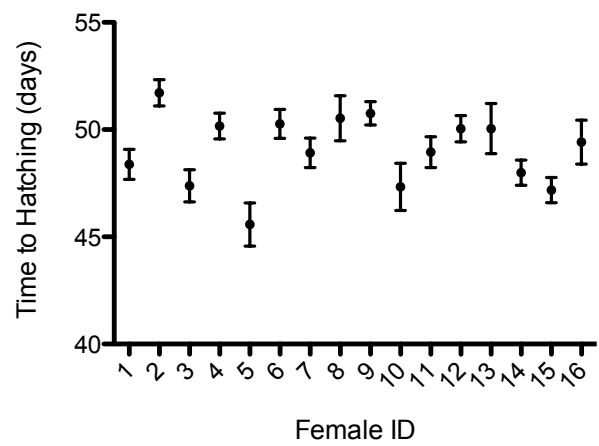

**D.**

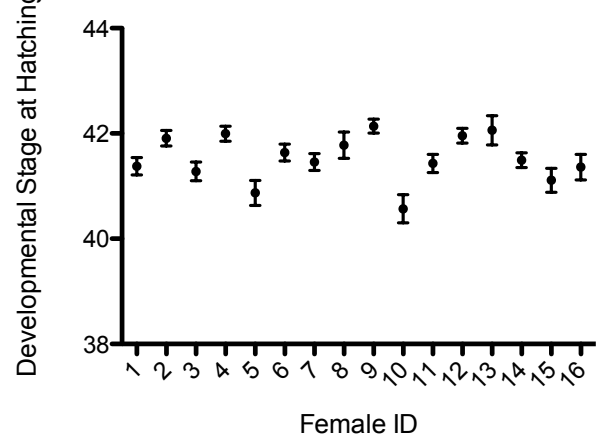

**E.**

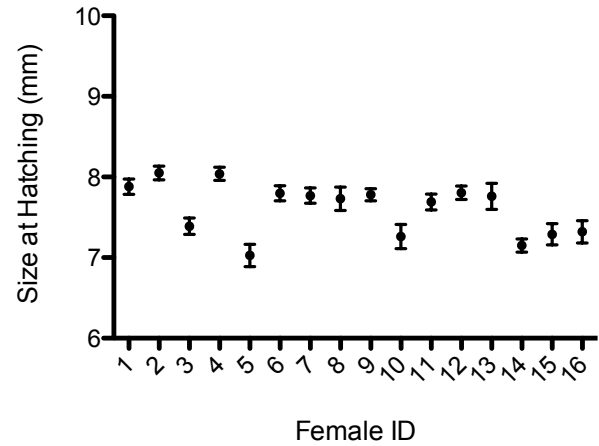

Supplement: Supplementary file 1 [file eva0006-0384-SD1.pdf]

## Figure 2

### Supplemental Information

A.

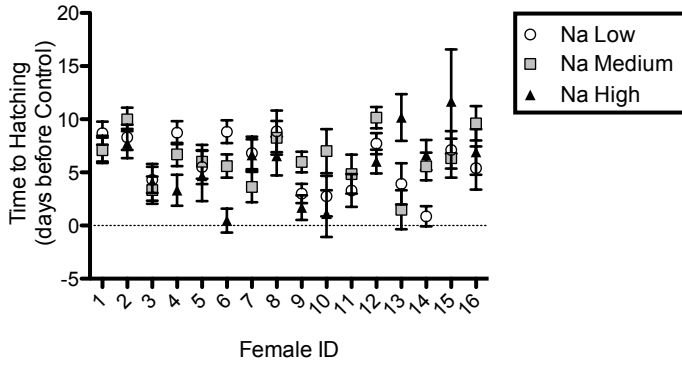

B.

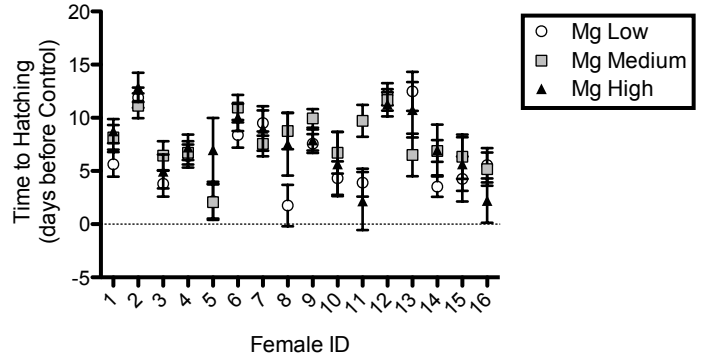

C.

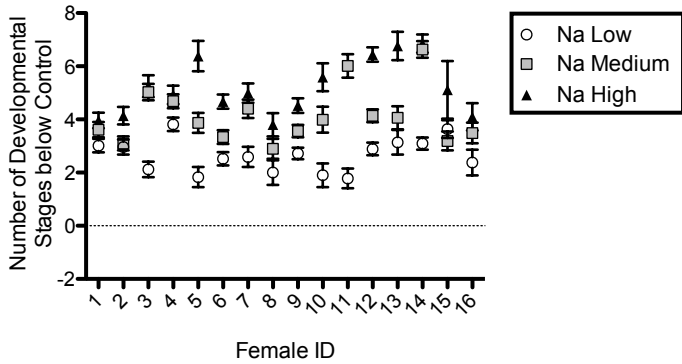

D.

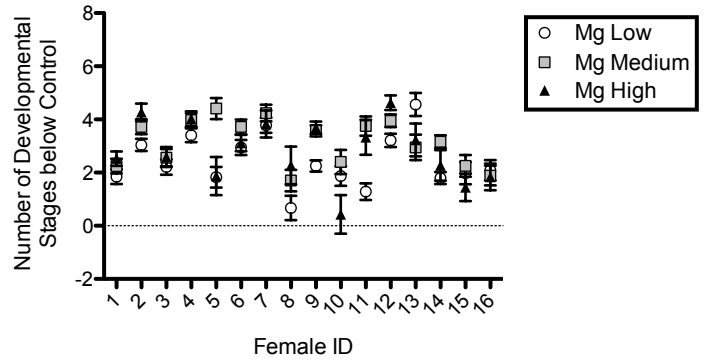

E.

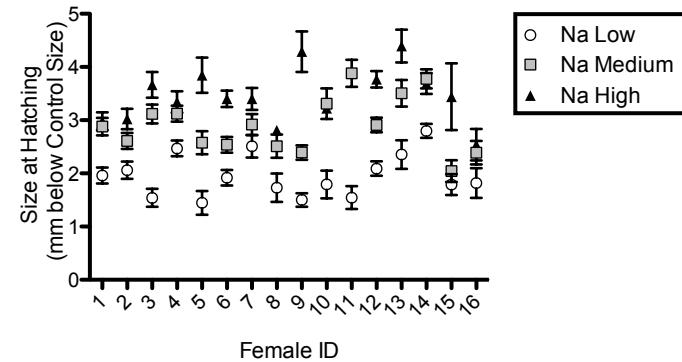

F.

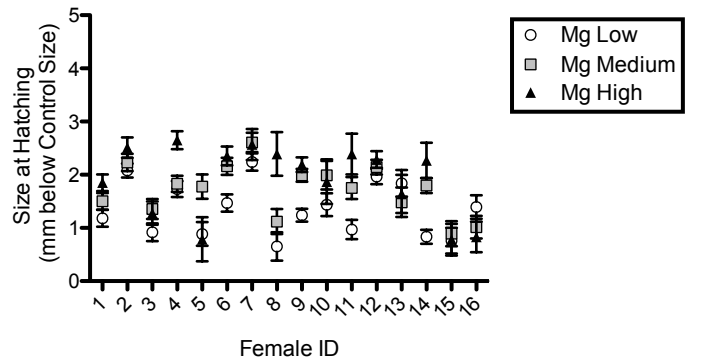

Supplement: Supplementary file 4 [file eva0006-0384-SD2.pdf]

**Fig 3.**  
**Supplemental**  
**Information**

**A.**

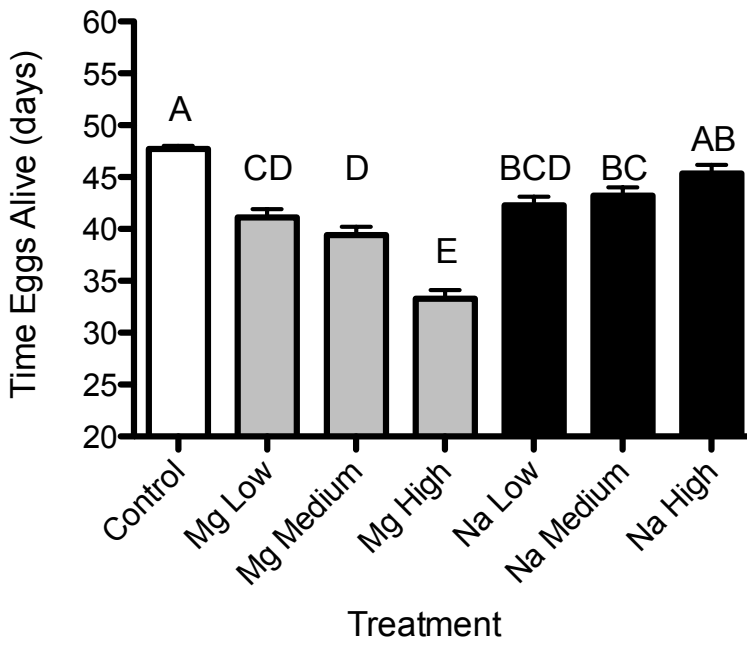

**B.**

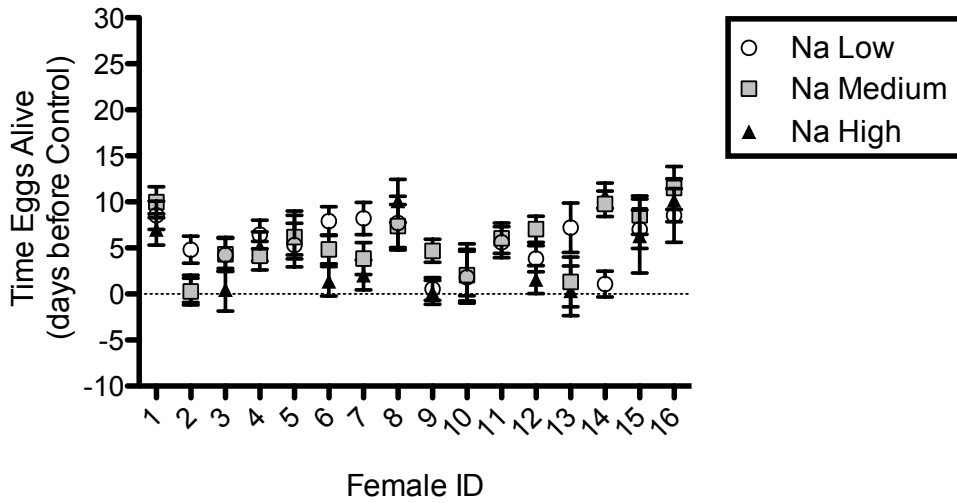

**C.**

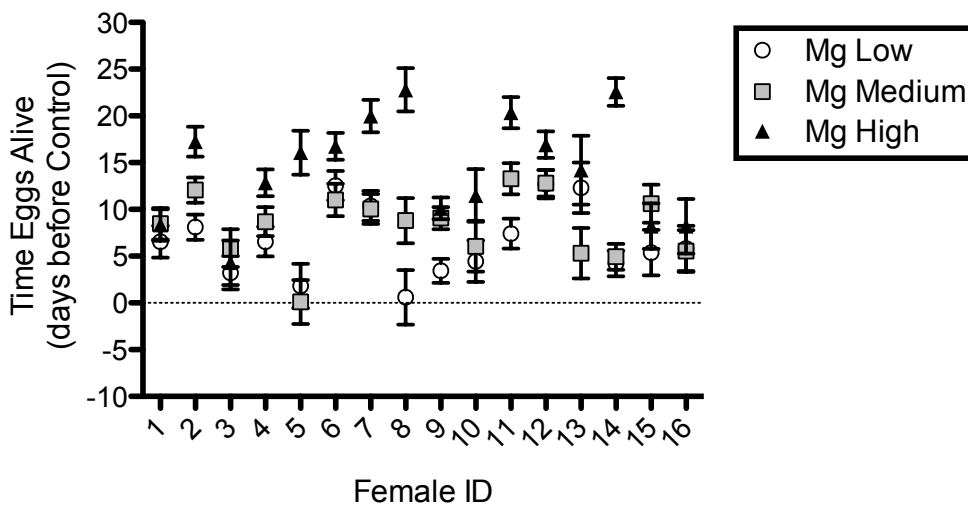

Supplement: Supplementary file 6 [file eva0006-0384-SD3.pdf]
