# Supplementary material for: Potential for local adaptation in response to an anthropogenic agent of selection: effects of road deicing salts on amphibian embryonic survival and development
Source: Evol Appl. 2012 Oct 1;6(2):384–92. doi: 10.1111/eva.12016 (PMC3586626; doi:10.1111/eva.12016)
Supplement: Supplementary file 2 [file eva0006-0384-SD5.docx]

**Figure Legends – Supplemental Information**

**Fig 1-SupplInfo.** The interfamily variation in **A.** Percentage of eggs survived, **B.** Time eggs survived (days), **C.** Time to hatching (days), **D.** Developmental stage at hatching, and **E.** Size (length) at hatching (mm) for the offspring of 16 different female newts raised in Control solution. Data are mean (±SE) values for all of a specific female’s offspring raised in that Control treatment. There is significant variation among females for all metrics (A-D) examined. Figures C-E reprinted from Hopkins *et al*. (2012).

**Fig 2-SupplInfo.** Significant variation in the response of eggs from different females to NaCl (A,C,E) and MgCl_2_ (B,D,F) salt concentrations for **A,B.** Time to hatching (days before eggs hatching in Control), **C.D.** Developmental stage at hatching (number of developmental stages below larvae hatching in Control), and **E.F.** Size (total length in mm) at hatching (mm below the size of larvae hatching in control).

**Fig 3-SupplInfo.** The effect of salinity concentration (A) and maternal identity (B,C) on time eggs survived (days) raised under increasing concentrations of NaCl (A,B) and MgCl_2_ (A,C). **A.** There is a significant effect of salt treatment on the mean (±SE) number of days eggs survived. Different letters indicate significant differences between treatments (Tukey-adjusted multiple comparisons). **B.** Mean (±SE) number of days (before those eggs in Control solution) eggs survived in three increasing concentrations of NaCl for 16 different female newts. There is significant variation in the response of eggs from different females to the treatments (Table 1 for detailed statistics). **C.** Same results for three increasing concentrations of MgCl_2_.
