# Supplementary material for: Potential for local adaptation in response to an anthropogenic agent of selection: effects of road deicing salts on amphibian embryonic survival and development
Source: Evol Appl. 2012 Oct 1;6(2):384–92. doi: 10.1111/eva.12016 (PMC3586626; doi:10.1111/eva.12016)
Supplement: Supplementary file 8 [file eva0006-0384-SD4.docx]

**Table 3.** Variation among families in (a) egg survival, (b) time to hatching (c) stage at hatching and (d) size (length) at hatching at different treatments, reporting the mean, minimum and maximum values (mean ± SE) for families for each treatment.

| Treatment | | Mean | | Min | | *N* | Max | | | *N* |
| --- | --- | --- | --- | --- | --- | --- | --- | --- | --- | --- |
| a. Egg Survival (%) | | | | | | | | | | |
| Control | | 91.50 ± 1.86 | | 73.98 ± 2.73 | | 321 | 97.96 ± 1.78 | | | 78 |
| Na Low | | 82.87 ± 3.53 | | 50.00 ± 11.34 | | 25 | 96.01 ± 5.77 | | | 14 |
| Mg Low | | 85.78 ± 2.15 | | 66.56 ± 10.43 | | 25 | 97.23 ± 3.98 | | | 21 |
| Na Medium | | 76.76 ± 3.90 | | 36.15 ± 10.75 | | 25 | 95.34 ± 6.76 | | | 12 |
| Mg Medium | | 75.99 ± 3.57 | | 50.00 ± 11.34 | | 24 | 96.79 ± 4.61 | | | 18 |
| Na High | | 53.31 ± 4.92 | | 2.88 ± 4.13 | | 20 | 83.18 ± 8.12 | | | 26 |
| Mg High | | 54.54 ± 6.08 | | 17.17 ± 8.44 | | 25 | 97.56 ± 3.50 | | | 24 |
|  | | | | | | | | | | |
| b. Time Eggs Alive (days) | | | | | | | | | | |
| Control | 47.72 ± 0.46 | | 44.67 ± 0.75 | | 321 | | | 49.87 ± 1.73 | 58 | |
| Na Low | 42.29 ± 0.56 | | 38.00 ± 2.71 | | 24 | | | 46.02 ± 2.23 | 35 | |
| Mg Low | 41.12 ± 0.82 | | 35.82 ± 2.58 | | 26 | | | 48.78 ± 4.43 | 9 | |
| Na Medium | 43.22 ± 1.17 | | 35.13 ± 2.23 | | 35 | | | 51.58 ± 3.83 | 12 | |
| Mg Medium | 39.43 ± 0.84 | | 34.67 ± 2.71 | | 24 | | | 45.50 ± 3.83 | 12 | |
| Na High | 46.89 ± 1.55 | | 34.24 ± 2.19 | | 38 | | | 59.50 ± 3.83 | 12 | |
| Mg High | 33.31 ± 1.47 | | 22.36 ± 2.31 | | 33 | | | 42.49 ± 3.49 | 14 | |
|  | | | | | | | | | | |
| b. Time to Hatching (days) | | | | | | | | | | |
| Control | | 49.04 ± 0.41 | | 45.58 ± 1.01 | | 79 | 51.72 ± 0.61 | | | 232 |
| Na Low | | 43.16 ± 0.63 | | 39.69 ± 1.78 | | 23 | 47.74 ± 1.46 | | | 32 |
| Mg Low | | 42.58 ± 0.71 | | 37.56 ± 3.01 | | 9 | 48.78 ± 3.01 | | | 9 |
| Na Medium | | 43.44 ± 0.80 | | 39.80 ± 2.64 | | 11 | 51.58 ± 2.60 | | | 12 |
| Mg Medium | | 41.50 ± 0.57 | | 38.36 ± 1.62 | | 28 | 47.66 ± 2.67 | | | 10 |
| Na High | | 41.47 ± 2.96 | | 35.50 ± 5.49 | | 2 | 52.45 ± 1.70 | | | 21 |
| Mg High | | 42.25 ± 0.97 | | 38.59 ± 4.00 | | 4 | 52.99 ± 4.13 | | | 3 |
|  | | | | | | | | | | |
| c. Stage at Hatching | | | | | | | | | | |
| Control | | 41.53 ± 0.11 | | 40.57 ± 0.27 | | 60 | 42.14 ± 0.13 | | | 238 |
| Na Low | | 38.88 ± 0.14 | | 37.47 ± 0.56 | | 14 | 39.78 ± 0.71 | | | 9 |
| Mg Low | | 39.10 ± 0.22 | | 37.5 ± 0.71 | | 9 | 41.11 ± 0.71 | | | 9 |
| Na Medium | | 37.40 ± 0.29 | | 34.86 ± 0.45 | | 21 | 38.89 ± 0.63 | | | 11 |
| Mg Medium | | 38.37 ± 0.22 | | 36.46 ± 0.63 | | 10 | 40.08 ± 0.66 | | | 9 |
| Na High | | 34.09 ± 2.29 | | 34.49 ± 0.81 | | 5 | 37.98 ± 0.69 | | | 7 |
| Mg High | | 38.69 ± 0.20 | | 37.33 ± 0.41 | | 22 | 40.14 ± 0.99 | | | 3 |
|  | | | | | | | | | | |
| d. Size at Hatching (mm) | | | | | | | | | | |
| Control | | 7.61 ± 0.08 | | 7.03 ± 0.14 | | 79 | 8.05 ± 0.08 | | | 191 |
| Na Low | | 5.65 ± 0.11 | | 4.35 ± 0.21 | | 30 | 6.28 ± 0.20 | | | 32 |
| Mg Low | | 6.26 ± 0.10 | | 5.53 ± 0.26 | | 21 | 7.08 ± 0.41 | | | 9 |
| Na Medium | | 4.70 ± 0.15 | | 3.37 ± 0.26 | | 21 | 5.44 ± 0.23 | | | 24 |
| Mg Medium | | 5.88 ± 0.12 | | 5.16 ± 0.28 | | 15 | 6.61 ± 0.38 | | | 9 |
| Na High | | 3.95 ± 0.31 | | 3.18 ± 0.47 | | 5 | 5.03 ± 0.27 | | | 15 |
| Mg High | | 5.69 ± 0.12 | | 4.88 ± 0.41 | | 6 | 6.51 ± 0.43 | | | 7 |
